# Laparoscopic Resection of a Colovesical Fistula Secondary to Diverticulitis Using Near-Infrared Fluorescent Ureteral Catheters

**DOI:** 10.70352/scrj.cr.25-0829

**Published:** 2026-04-29

**Authors:** Yoki Endo, Yuya Tanaka, Risa Imagawa, Tatsunori Suzuki, Moe Saito, Shimpei Takagi, Katsushi Suenaga, Taihei Soma, Takahiro Ozaki, Tsunehisa Matsushita, Yasuhiro Sumi

**Affiliations:** 1 Department of Surgery, NHO Shizuoka Medical Center, Sunto, Shizuoka, Japan; 2 Imperial College London, White City Campus, London, UK

**Keywords:** near-infrared fluorescent ureteral catheters, minimally invasive surgery, colovesical fistula, sigmoid diverticulitis

## Abstract

**INTRODUCTION:**

A colovesical fistula is a rare complication most often caused by diverticulitis, typically requiring surgical repair due to the pressure gradient in which the intraluminal pressure within the colon exceeds that of the bladder.

**CASE PRESENTATION:**

We report a 49-year-old man with pneumaturia and fecaluria secondary to chronic sigmoid diverticulitis. Imaging demonstrated a colovesical fistula, and cystoscopy confirmed fecal discharge at the bladder dome. Laparoscopic sigmoidectomy with bladder repair was performed after preoperative placement of bilateral near-infrared fluorescent ureteral catheters (NIRFUCs). Intraoperatively, dense adhesions were encountered, but the ureters were instantly and clearly identified with fluorescence prior to and throughout dissection, preventing iatrogenic injury. The fistula was resected, the bladder defect closed, and colorectal anastomosis completed without diversion. Operative time was 360 min with minimal blood loss, and recovery was uneventful. Histopathological examination confirmed diverticulitis without malignancy.

**CONCLUSIONS:**

This case highlights the feasibility of laparoscopic management of colovesical fistula and demonstrates the usefulness of NIRFUCs in ensuring ureteral safety during challenging pelvic surgery, especially in the era of minimally invasive surgery where fluorescence compensates for the lack of tactile feedback.

## Abbreviations

ICG
: indocyanine green
NIRFUC
: near-infrared fluorescent ureteral catheter

## INTRODUCTION

A colovesical fistula is a rare complication most often caused by diverticulitis, typically requiring surgical repair because the intraluminal pressure of the colon exceeds that of the bladder. As the global population continues to age, the number of surgically managed cases of colovesical fistulas secondary to colon diverticulitis is expected to increase. Recently, laparoscopic surgery has been widely used in gastrointestinal surgery, but the procedures for cases involving severe inflammation are technically challenging, and the open conversion rate is still high.^1)^ One reason is that severe inflammation often obscures the course of the ureter, which is an organ at risk of injury during colorectal surgery. NIRFUCs, which have been approved for clinical application and are insurance-covered with benign disease as a labeled indication in Japan, can be easily inserted using the same technique as conventional ureteral stents and greatly aid in real-time navigation of the ureter during surgery.

Herein, we report the case of a male patient with a colovesical fistula secondary to sigmoid colon diverticulitis who underwent laparoscopic surgery with visualization of the ureters using the NIRFUCs.

## CASE PRESENTATION

**Fig. 1:**
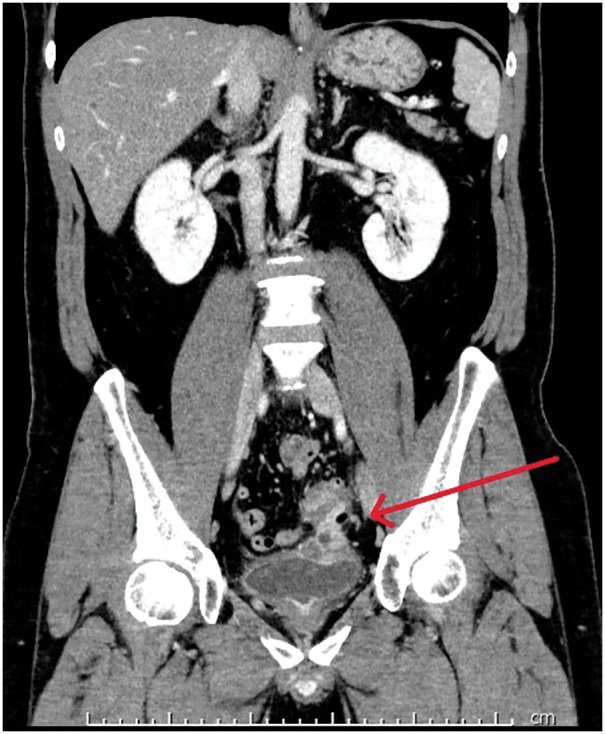
CT showed multiple diverticula in the sigmoid colon with increased density of surrounding fatty tissue. A colovesical fistula was formed. The arrow indicates the fistula that contacts the bladder dome, and the contrast enhancement extends continuously to the bladder mucosal surface. A small amount of air was noted within the bladder.

**Fig. 2:**
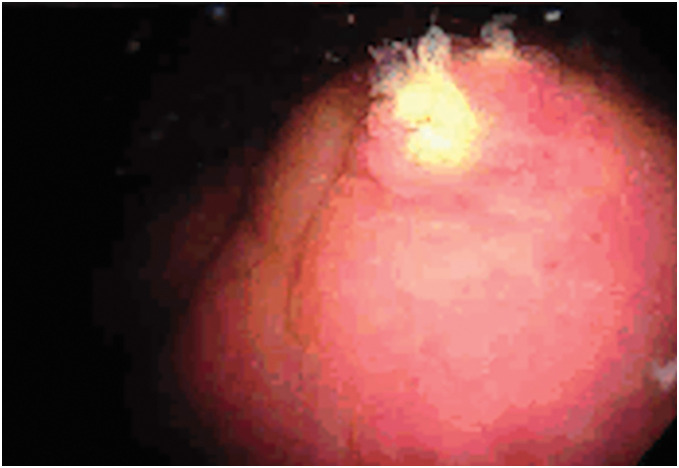
Cystoscopy revealed a protrusion at the bladder dome, with visible fecal discharge from the lesion.

A 49-year-old man presented with pneumaturia and fecaluria for 2 weeks. He was referred to our hospital with a diagnosis of a colovesical fistula secondary to chronic sigmoid diverticulitis. His medical history included hypertension and prior episodes of sigmoid diverticulitis. Physical examination revealed a flat, soft abdomen without tenderness. Laboratory findings were unremarkable. Contrast-enhanced CT showed multiple diverticula in the sigmoid colon with surrounding fat stranding (**Fig. 1**). The sigmoid colon was in contact with the bladder dome, and contrast enhancement of the sigmoid colon appeared continuous with the bladder mucosa. A small amount of intravesical air was also noted. Colonoscopy confirmed the presence of multiple diverticula, but the fistula could not be identified. Cystoscopy revealed a protrusion at the bladder dome, with visible fecal discharge from the lesion, which was distant from both ureteral orifices (**Fig. 2**). Laparoscopic sigmoidectomy was planned for definitive treatment. On the day of surgery, bilateral straight-type NIRFUCs were placed cystoscopically by a urologist, under general anesthesia. The insertion required approximately 15 min.

**Fig. 3:**
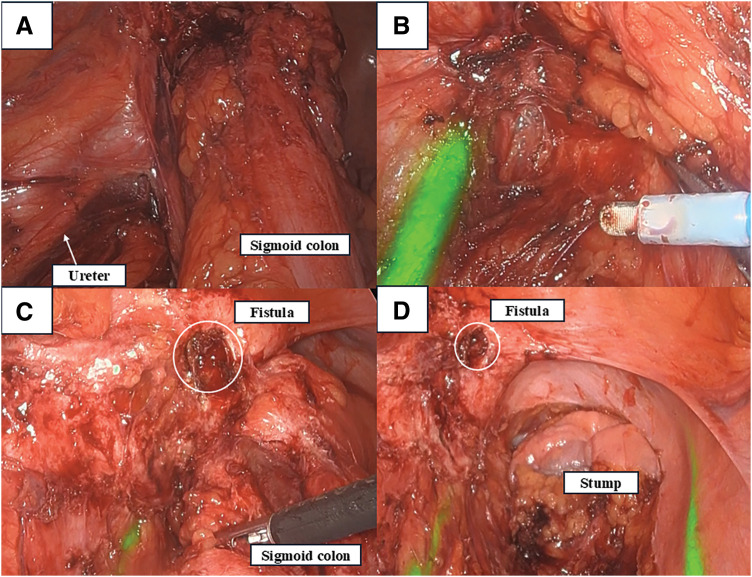
(**A**) Normal light observation. Beyond this point, dissection was difficult due to inflammatory adhesions, and the course of the ureter was unclear. (**B**) Enlarged view of (**A**) with fluorescence observation. Only a moment was required to identify the course of the ureters, allowing for safe and easy dissection. (**C**) After the fistula resection. The area around the fistula (circle) was the most severely inflamed. (**D**) After the sigmoid colon resection. The ureters were clearly visible under fluorescence observation.

Intraoperative findings revealed that the sigmoid colon and the bladder were densely adherent due to chronic inflammation. The mobilization of the sigmoid colon was performed while preserving the inferior mesenteric artery. Under standard white-light imaging, beyond the level of the left common iliac artery, identification of the distal left ureter became extremely difficult due to thick scar tissue associated with chronic inflammation. The NIRFUCs allowed instant and clear visualization of the ureters prior to and throughout dissection, preventing iatrogenic injury (**Fig. 3**). While the retroperitoneal plane itself was generally preserved, the ventral aspect and the left lateral plane were obscured by inflammatory scarring. In these areas, where normal anatomical planes were not clearly maintained, we proceeded with careful dissection while intermittently using the fluorescence mode to confirm ureteral integrity. The size of the fistula was only a few millimeters, so partial cystectomy was not necessary. After dissecting the fistula, the bladder was filled with saline via a urethral catheter, but no leakage was observed from the fistula. The fistula was located at the bladder dome and closed using a 4-0 absorbable running suture in 1 layer. The sigmoid colon was extracted through a small abdominal incision, the mesentery was dissected along the bowel, and the specimen was removed. The colorectal anastomosis was completed using a double-stapling technique, and a leak test was performed and confirmed to be negative. Considering the patient was a young man with no significant comorbidities, no contamination within the abdominal cavity, and no signs of inflammation at the anastomotic site, a diverting stoma was not created. The operative time, including the time required to place the NIRFUCs, was 360 min, and total blood loss was 40 mL. Histopathological examination of the resected colon revealed multiple diverticula with infiltration of inflammatory cells, but no evidence of malignancy. Oral intake was resumed on POD 3. Both the urethral catheter and ureteral catheters were left in place for bladder decompression and removed on POD 7 following confirmation of no leakage on cystography. The postoperative course was uneventful with no catheter-related complications, and the patient was discharged on POD 11.

## DISCUSSION

A colovesical fistula is a rare complication of inflammatory, neoplastic, traumatic, or iatrogenic disease. Diverticulitis is the most common etiology, accounting for 87.9% of cases.^2)^ Spontaneous closure is rare due to the high intraluminal pressure gradient, and surgical intervention is usually required. As a surgical option, a 1-stage procedure involving partial colectomy and partial cystectomy is often performed. However, in cases where acute inflammation is extensive, a 2- or 3-stage approach is generally recommended, in which a diverting stoma is first created to allow complete resolution of the inflammation before definitive surgery.

In addition, the best surgical approach is still under debate. Regardless of the surgical approach, complete resection of the fistula is of paramount importance, as it is associated with favorable long-term outcomes. Open surgery allows definitive resection and bladder repair and may offer a safety advantage with respect to ureteral preservation, as the ureters can be directly palpated. However, it is associated with greater invasiveness. Laparoscopic surgery offers reduced postoperative pain and faster return to daily activity, yet it demands advanced technical expertise. Robotic surgery provides superior dexterity, 3D visualization, and potentially lower conversion rates than laparoscopy, especially in difficult dissections, but it entails significantly longer operative times and substantially higher costs. Its long-term advantages remain to be confirmed by large randomized studies.^3)^ Minimally invasive surgeries, during which surgeons greatly rely on visual information, put the ureters at risk of injury, especially in the presence of severe inflammatory changes that obscure anatomical landmarks. In recent years, laparoscopic surgery has become commonplace, but a large cohort study involving 111 laparoscopic cases of colovesical fistulas showed a high open conversion rate of 34.7%.^4)^ One of the reasons raised by the authors is the difficulty in identification of the ureters due to severe inflammation and dense fibrosis. In our case, NIRFUCs were available and allowed reliable intraoperative identification of the ureter. However, if they had not been available or identifying the ureters had been difficult, conversion to open surgery might have been required.

Various methods have been proposed to prevent ureteral injury during pelvic surgery. ICG infusion via a ureteral stent allows ureteral visualization with limited manipulation but suffers from weak fluorescence. Intravenous methylene blue avoids ureteral instrumentation, yet it also has low brightness. Lighted ureteral stents can illuminate the ureter effectively but are associated with significant risks, including thermal damage, obstruction, and stricture formation, limiting their popularity. In contrast, NIRFUCs incorporate a fluorescent resin that provides approximately 30-fold stronger signal than ICG.^5)^ NIRFUCs emit fluorescence from the catheter, rather than from the fluorescent dye in the urine; hence, the fluorescence is not affected by the flow of urine and is uniform and constant. Therefore, NIRFUCs offer intraoperative guidance, improving orientation and enabling safe dissection.

When ureteral injuries occur, prompt recognition and treatment are crucial. This may state the obvious, but ureteral injuries are difficult to detect intraoperatively, and approximately 25%–62% of patients are diagnosed postoperatively. In particular, laparoscopic surgery was reported to be a risk factor for delayed recognition of ureteral injury.^6)^ If not detected and treated promptly, ureteral injuries can have serious complications, including ureteral strictures and long-term renal function loss. The single greatest prognostic factor for ureteral injury is the time to diagnosis, and excellent outcomes are associated with intraoperative diagnosis and repair^7)^. We believe that a method to preserve the ureters is needed, and NIRFUCs can be 1 feasible option to both mitigate the risk and recognize ureteral injuries by real-time navigation of the ureter. Despite its considerable utility, only a few cases have been reported where NIRFUCs were used for colovesical fistulas secondary to colon diverticulitis^8–10)^ (**Table 1**). Ureteral preservation should be pursued to a greater degree in benign disease because the only barrier is its technical feasibility, which can be assisted with NIRFUCs. In contrast, existing literature describes its use in colorectal cancer, but dissection of the ureter is oncologically unadvised and may favor combined resection in locally advanced cases invading the ureter. Except for 1 case with missing data, NIRFUCs were inserted on the day of surgery. While this procedure may prolong anesthesia time, it is expected to contribute to shorter overall surgery times. Regarding visibility of ureters, whether the ureters could be identified prior to dissection appeared to vary depending on the degree of inflammation and the amount of visceral fat. In all cases, including ours, the surrounding inflammatory findings were severe, and the surgical duration varied depending on operative findings. However, no open surgery was required, and the procedures were completed safely without ureteral injury. A key limitation of this review is the presence of missing data in the small number of analyzed cases.

**Table 1:** Reported cases of colovesical fistula resected using NIRFUCs

No.	Author	Age	Sex	Surgical procedure	Operative time (min)	Timing of NIRFUC insertion	Catheter placement site	Visibility of the ureters	Diverting stoma	Complications
1	Osumi et al.^8)^	82	M	LAR (3-stage: colostomy, LAR, colostomy closure)	318	Day of surgery	n/a	Prior to dissection	Transverse colostomy	None
2	Okamoto et al.^9)^	40	M	LS	281	Day of surgery	Bilateral	During dissection	None	None
3	Tamura et al.^10)^	35	F	LS	n/a	n/a	Left	During dissection	None	None
4	Present case	49	M	LS	360	Day of surgery	Bilateral	Prior to dissection	None	None

F, female; LAR, laparoscopic anterior resection; LS, laparoscopic sigmoidectomy; M, male; min, minutes; n/a, not available; NIRFUC, near-infrared fluorescent ureteral catheter

## CONCLUSIONS

In the future, the number of laparoscopic and robotic procedures is expected to further increase. NIRFUCs have the potential to compensate for the inherent limitation of these minimally invasive approaches—the lack of tactile feedback—by providing clear and reliable ureteral visualization.

## DECLARATIONS

### Availability of data and materials

The datasets used and/or analyzed during the current study are available from the corresponding author on reasonable request.
